# Incidence and prevalence of multiple sclerosis during eras of evolving diagnostic criteria—a nationwide population-based registry study over five decades

**DOI:** 10.1177/20552173251326173

**Published:** 2025-03-16

**Authors:** Anna Maunula, Sini M Laakso, Matias Viitala, Merja Soilu-Hänninen, Marja-Liisa Sumelahti, Sari Atula

**Affiliations:** Translational Immunology Research Program, 3835 University of Helsinki, Helsinki, Finland; HUS Neurocenter, Department of Neurology, Hyvinkää Hospital, Hyvinkää, Finland; Translational Immunology Research Program, 3835 University of Helsinki, Helsinki, Finland; HUS Neurocenter, Helsinki University Hospital, Helsinki, Finland; StellarQ Ltd, Turku, Finland; Clinical Neurosciences, 8058 University of Turku, Finland; Neurocenter, Turku University Hospital, Turku, Finland; Faculty of Medicine and Health Technology, Tampere University, Tampere, Finland; HUS Neurocenter, 159841 Helsinki University Hospital, Helsinki, Finland; Department of Clinical Neurosciences, 3835 University of Helsinki, Helsinki, Finland

**Keywords:** Multiple sclerosis, MS, incidence, prevalence, epidemiology, diagnostic criteria

## Abstract

**Background:**

Impact of changing diagnostic criteria for the population-based incidence of multiple sclerosis (MS) has not been investigated.

**Objective:**

To assess the effect of changing diagnostic criteria on national MS incidence and prevalence in Finland from 1974 to 2021.

**Methods:**

We identified patients with MS (pwMS) through the National MS registry and the national Care Register for Healthcare and divided them into four groups based on the year of MS diagnosis: 1) Schumacher criteria (1974–1982), 2) Poser criteria (1983–2000), 3) Earlier McDonald criteria (2001–2016), and 4) Current McDonald criteria (2017–2021). Age-adjusted incidence and prevalence were calculated.

**Results:**

Age-adjusted incidence per 10^5^ person years increased from 3.7 (95% CI 3.5–3.8) during the Schumacher criteria period to 9.2 (95% CI 9.0–9.4) during the earlier McDonald criteria. During the Current McDonald criteria incidence stabilized to 8.6 (95% CI 8.3–9.0). Prevalence increased from 24.3 (95% CI 22.8–25.8) to 241.5 (95% CI 237.3–245.6) per 10^5^ person years.

**Conclusion:**

Both incidence and prevalence of MS increased significantly. Incidence showed a sharp increase when entering the twenty-first century, after which it stabilized. Increasing incidence was likely related to incorporation of MRI in the diagnostic criteria. Current diagnostic criteria did not further increase the incidence.

## Introduction

Multiple sclerosis (MS) is an immune-mediated disease of the central nervous system and one of the leading causes of neurological disability in young adults.^1^ The prevalence of MS has increased globally from 1960 to date^2–4^ with rising prevalence recorded in all regions of the world.^4^ Data from the twentieth century, from several geographical areas, show increased incidence of MS,^3,5–8^ whereas incidence in the late twentieth and early twenty-first century seems to become stable^9–12^ Highest age-standardized prevalence rates have been reported for North America, Western Europe and Australasia.^2^ A recently published systematic review, covering the published peer-reviewed literature on the change in worldwide MS incidence over time, challenged the concept of a global increase in the incidence of MS over the past three decades;^13^ research protocols using consistent criteria for patient identification and with high population coverage showed stable trends, whereas increases were seen in studies limited in these aspects.

Nationwide prevalence of MS in Finland has not been studied since the 1960's.^14^ The focus of studies has been on regional occurrence, particularly in high-risk western parts of Finland.^5,7,8,15–22^ Recent occurrence updates in the twenty-first century support the observation that Finland is a high-risk area of MS.^7,15,22^ They also support the observation of an East-West gradient, made originally in 1966, that Western Finland has a higher prevalence of MS compared to Eastern Finland.^14,22^ This may suggest specific genetic susceptibility for MS, although environmental factors can also be at play.^23–25^

The Schumacher criteria in 1965 ^26^ and the Poser criteria in 1983 ^27^ relied mainly on patient-reported symptoms and neurological findings, supported by increased immunological activity in cerebrospinal fluid (CSF) and possible alterations in neurophysiological and/or urological testing . MRI criteria were first included in the McDonald criteria in 2001.^28^ Since then, the criteria have been updated several times.^29,30^ The most recent update in 2017 ^31^ enabled diagnosis after the first demyelinating event, if MRI criteria for dissemination in space are fulfilled and criteria for dissemination in time (DIT) are fulfilled or oligoclonal bands are detected in the CSF. This was a significant change to earlier criteria where two relapses or further MRI evidence for DIT were required.

Applying 2017 McDonald criteria has been shown to increase the number of patients receiving diagnosis at the time of first demyelinating event.^32–36^ Studies show higher sensitivity (68–100%) but also lower specificity (14–53%) for McDonald 2017 criteria when compared to 2010 McDonald criteria in predicting the second clinical event.^34,37–40^ However, with the likelihood of a second event influenced by early initiation of a DMT, and the findings of a similar area under the curve suggesting capture of the same patient population in follow-up, accuracy of 2017 McDonald criteria has been perceived as similar^34,38^ or higher^35,37^ in comparison to 2010 criteria. Sustained accuracy has also been suggested when comparing 2010 McDonald criteria to 2005 criteria.^36^

Population-based studies on the effect of evolving criteria on the incidence of MS and the accompanying changes in prevalence have, to our knowledge, not been conducted. We aimed to examine the changes in the incidence and prevalence of MS in Finland from 1974 to 2021, using the eras set by altering diagnostic criteria to evaluate their effect on the national incidence rate and to discover changes in prevalence.

## Methods

### Study cohort

This study was permitted by Finnish Social and Health Data Permit Authority (Findata), which authorizes secondary use of social and health care data in Finland and can combine information from different national registries.

We identified patients with MS (pwMS) diagnosed between 1974 and 2021. Patients were primarily found from the National MS registry.^41^ If a patient was not found in the MS registry data was collected from the National Care Register. All healthcare providers, both publicly and privately funded, are obliged by law to report information to the Care Register, including diagnosis according to ICD codes for all doctor's visits. Patients with at least two separate visits with at least 1 year apart with diagnosis code G35 from 1996 onwards and/or G340 in 1974–1995 were included in the study. Patients that had later received a diagnosis for a neuromyelitis optica spectrum disorder (ICD-10 G36.0, ICD-9 341.0) were excluded from the study.

**Figure 1.**
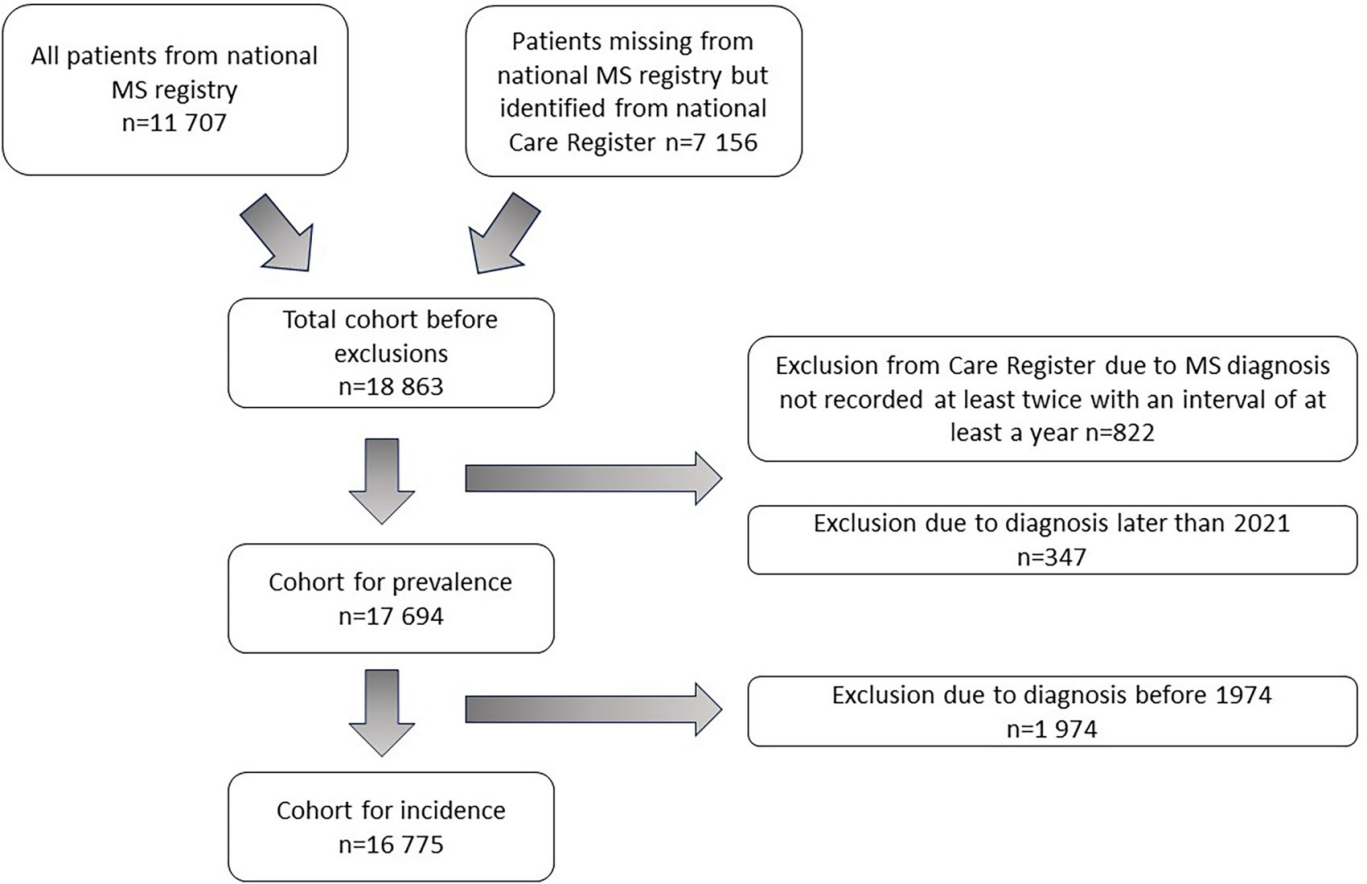
Flow chart of the process of forming the cohort.

Approximately 80% of the cohort diagnosed in the twenty-first century was identified from the MS-register, whereas the portion was significantly lower, approximately 43%, in the twentieth century. The registry was launched in 2014 is estimated to cover 85–90% of current Finnish pwMS and is described by Laakso et al.^41^ The patients not included in the MS-register were identified through a national Care Register for Healthcare (Figure 1).

Main source of MS diagnosis date was the date recorded in the MS registry. Diagnosis date for patients identified through Care Register was derived as first visit using ICD-10/ICD-9 codes G35/340. Information on the MS subtype (relapsing-remitting, progressive) was not included as it was not available for Care Register patients. We identified date of birth, gender, and place of residence at age of 15 years for all pwMS.

### Study intervals set by the evolving diagnostic criteria

Patients were allocated into four study subgroups based on the year of MS diagnosis:

1. Schumacher criteria (1974–1982)^26^
2. Poser criteria (1983–2000)^27^
3. Earlier McDonald criteria (2001–2016)^28–30^
4. Current McDonald criteria (2017–2021)^31^

The first time frame (1974–1982; Schumacher criteria) was limited to these years because data from Care Register is available from 1969 forwards and a 5-year run-in period was needed for incidence calculations.

### Statistical analysis

Data analysis and visualization were performed on pseudonymized data using RStudio (Version 2023.09.1). Partial dates were imputed as the middle of the month or year. Numerical variables were expressed as means with standard deviations or medians with interquartile ranges. Categorical variables were expressed as frequencies and proportions based on non-missing data.

Age-adjusted incidence and prevalence ratios were calculated based on yearly reported Finnish population statistics (Statistics Finland) and European Standard Populations (revision 1976 for diagnoses between 1974 and 2000 and revision 2013 for diagnoses between 2001 and 2021)^42^ using 5-year age periods. Standard population distributions were used as weights for each age-specific rate. Incidence analysis was done using diagnosis year subgroups summing population counts and newly diagnosed patients within each year interval. Prevalence analysis was done separately for each year. Analyses were separated for both sex categories. Analyzed ratios were reported with 95% confidence intervals based on Poisson approximation.

## Results

### Changes in incidence and prevalence

**Figure 2.**
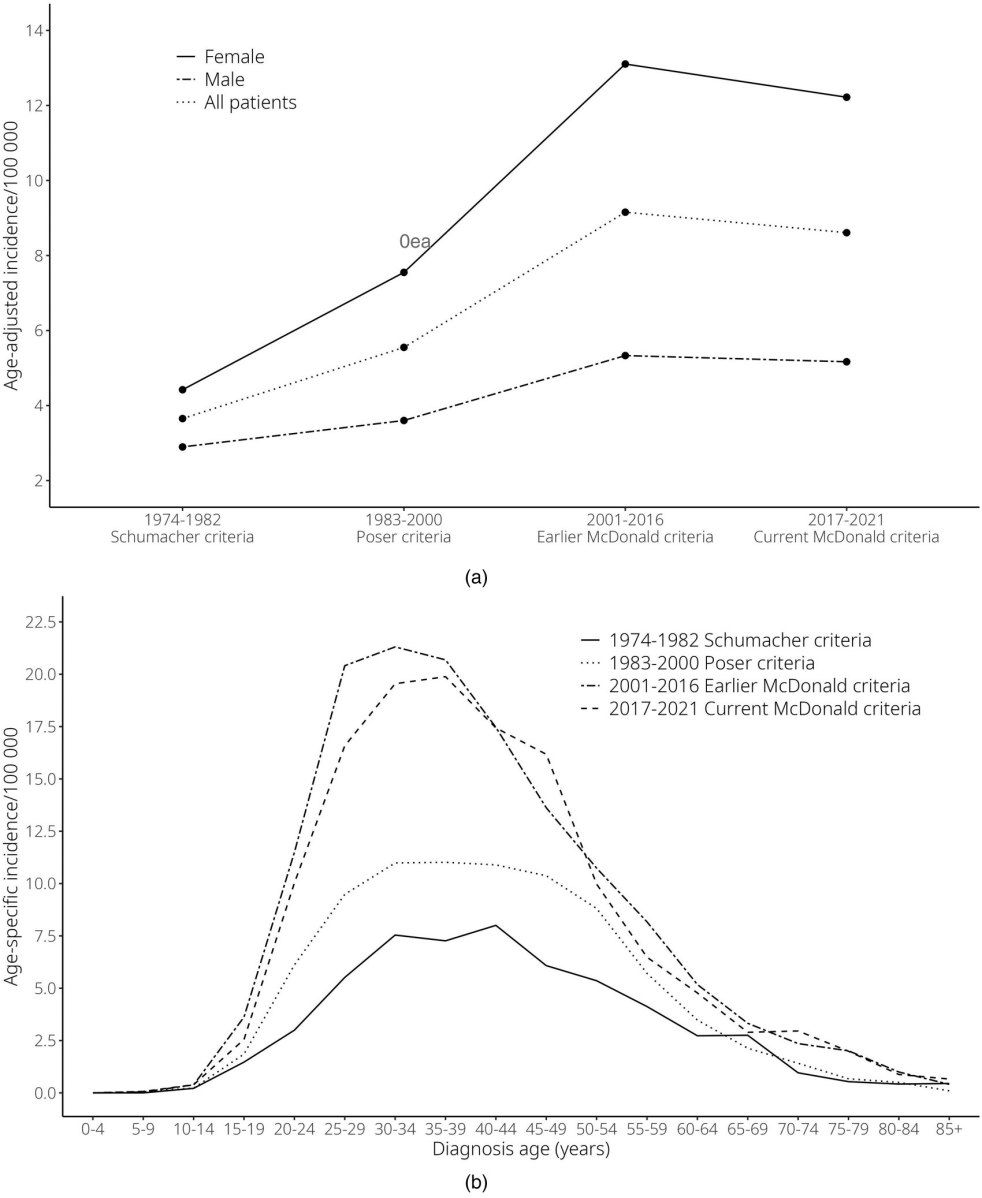
Incidence of MS during the diagnostic eras. Values are point estimates for each diagnostic era separately. (a) Age-adjusted incidence per 10^5^ person years by eras of diagnostic criteria*.* (b) Age-specific incidence per 10^5^ person years by age group at diagnosis shown for different eras of diagnostic criteria.

Both crude and age-adjusted incidence showed a sharp increase when entering the era of the earlier McDonald criteria (2001–2016) (Table 1). Age-adjusted incidence during the earlier McDonald criteria was 9.2/10^5^ person years (95% CI 8.3, 9.0) and during the current 2017 McDonald criteria 8.6/10^5^ person years (95% CI 8.9, 9.4), showing a slight decline (Figure 2(a)).

**Table 1.** Patient demographics with incidence and prevalence according to eras of diagnostic criteria.

	**Schumacher criteria (1974–1982) (*n*** **=** **1558)**	**Poser criteria (1983–2000) (*n*** **=** **5183)**	**Earlier McDonald criteria (2001–2016) (*n*** **=** **7758)**	**Current McDonald criteria (2017–2021) (*n*** **=** **2276)**
**Sex (Females); *n* (%)**	955 (61.3%)	3500 (67.5%)	5487 (70.7%)	1581 (69.5%)
**Age at MS diagnosis (years);** **Mean (SD)**	39.5 (12.9)	39.8 (12.1)	39.7 (13.1)	40.6 (13.4)
Females	39.4 (13.0)	39.7 (12.0)	39.4 (13.0)	39.7 (13.0)
Males	39.7 (12.9)	40.0 (12.2)	40.7 (13.2)	42.6 (13.9)
**Age at MS diagnosis (years);** **Median (Q1-Q3)**	37.5 (30.0–48.0)	39.0 (31.0–48.0)	38.0 (30.0–48.0)	39.0 (31.0–48.0)
Females	37.0 (30.0–48.0)	39.0 (31.0–48.0)	38.0 (29.0–48.0)	38.0 (30.0–47.0)
Males	38.0 (30.0–49.0)	39.0 (31.0–48.0)	39.0 (30.0–50.0)	41.0 (32.0–52.0)
**Age-adjusted incidence rate per 10^5^ person years (95% CI)**	3.7 (3.5–3.8)	5.6 (5.4–5.7)	9.2 (9.0–9.4)	8.6 (8.3–9.0)
Females (95% CI)	4.4 (4.1–4.7)	7.6 (7.3–7.8)	13.1 (12.8–13.5)	12.2 (11.6–12.8)
Males (95% CI)	2.9 (2.7–3.1)	3.6 (3.4–3.8)	5.3 (5.1–5.6)	5.2 (4.8–5.6)
**Incidence female/male ratio (95% CI)**	1.5 (1.4–1.7)	2.1 (2.0–2.2)	2.5 (2.3–2.6)	2.4 (2.2–2.6)
**Age-adjusted prevalence rate per 10^5^ person years (95% CI)** ^a^	24.3 (22.8–25.8)	111.3 (108.5–114.2)	218.4 (214.4–222.4)	241.5 (237.3–245.6)
Females (95% CI)^a^	27.9 (25.7–30.1)	151.2 (146.5–155.8)	309.7 (303.0–316.3)	344.2 (337.3–351.3)
Males (95% CI)^a^	20.7 (18.7–22.7)	71.6 (68.4–74.8)	127.0 (122.7–131.3)	138.8 (134.4–143.3)

a Age-adjusted prevalence rate in this table is from the years 1974, 2000, 2016 and 2021. SD: standard deviation.

When examining the effect of sex, an increased incidence during earlier McDonald criteria (2001–2016) was seen in both males and females, being more pronounced in females with age-adjusted incidence rate up to 13.1/10^5^ person years.

Age-specific distribution of incidence by age groups at diagnosis was similar throughout the study period (Figure 2(b)). A peak at 30–34 years was seen during earlier McDonald criteria (2001–2016), whereas it was slightly higher, at 35–39 years, during 2017 McDonald criteria. When dividing by sex, peak age at diagnosis in the twenty-first century was at a younger age for females (Supplementary Figure 1).

**Figure 3.**
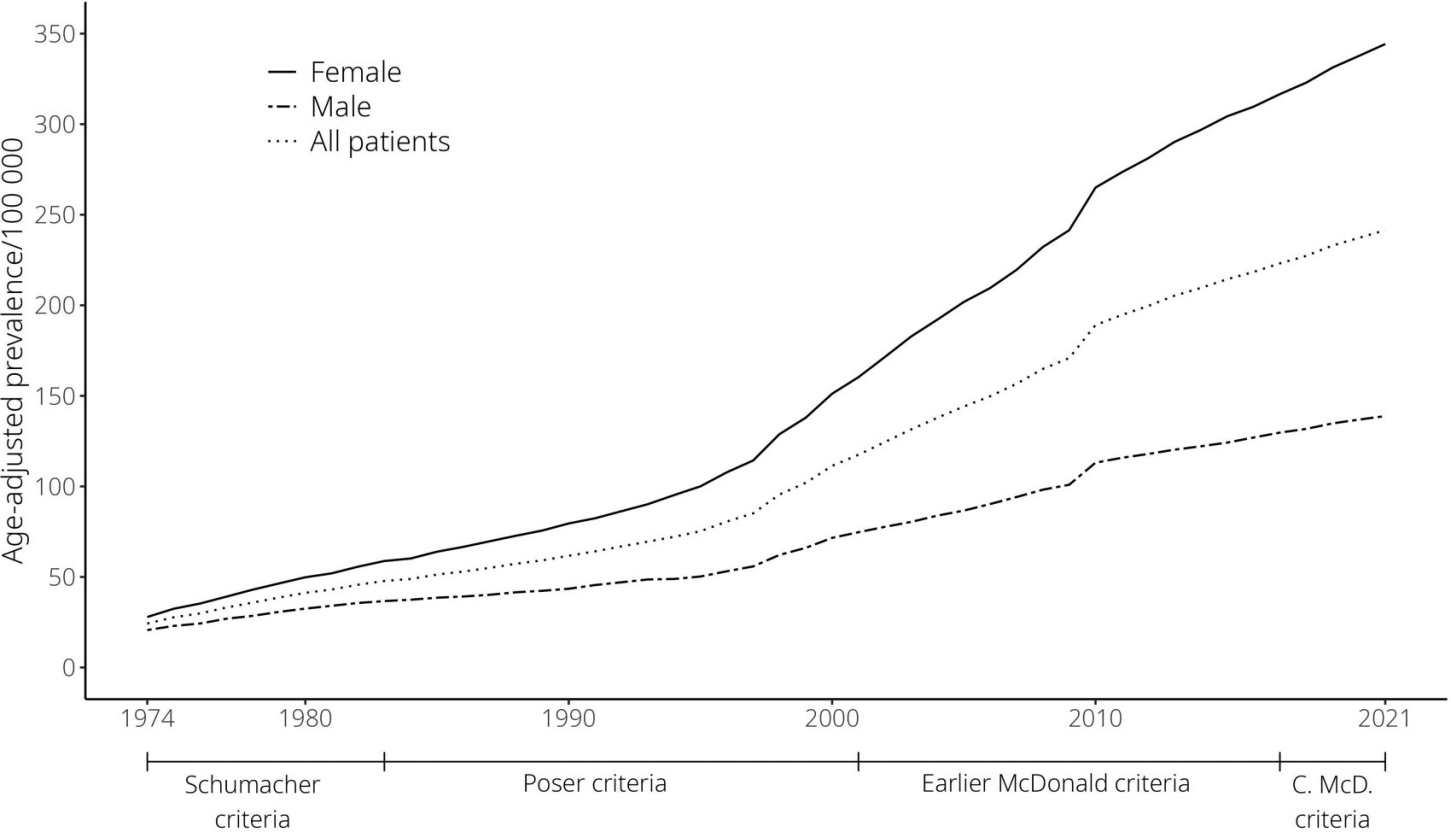
Trend of age-adjusted prevalence per 10^5^ person years from 1974 to 2021. C.McD. Criteria = Current McDonald Criteria.

When looking at age-adjusted prevalence, it showed a tenfold increase from 24.3 per 10^5^ person years in 1974 to 241.3 per 10^5^ person years in 2021 (Figure 3). This was mainly attributed to a steep rise in incidence in the late 1990s, which was more pronounced in females.

### Mean age at diagnosis and female-to-male ratio

For females, mean age at MS diagnosis was stable throughout the study period, around 40 years, whereas a rise was seen in males, from 39.7 during the Shumacher criteria to 42.6 during the current McDonald criteria (Table 1). Median age was slightly lower compared to mean age especially in the first period studied. Care Register for healthcare had higher mean diagnosis age throughout the study period ranging from 12.2 years higher during the first criteria period to 3,6 years higher in the last study period (Supplementary Table).

Female-to-male ratio (F/M-ratio) showed an increase during the entire study period from 1.5 to 2.4 (Table 1). The change was most prominent when entering from Schumacher to Poser criteria (1.5 to 2.1). Care register had a lower percentage of females compared to the national MS register (64% vs 71%).

## Discussion

Our nationwide analysis showed that both the incidence and prevalence of MS increased during the study period from 1974 to 2021 in Finland. After a sharp increase in incidence when entering the twenty-first century, rates showed a stabilizing trend, similar to what has been seen in other studies^9–12^

Incidence rate in the twenty-first century in this study was similar to other studies.^8,11,43^ The stabilizing, even slightly decreasing trend of incidence comparing earlier and current McDonald criteria indicates that the current diagnostic criteria are accurate and reflect the true incidence of MS. The increasing incidence of MS entering the twenty-first century is most likely due to changes in diagnostic criteria. Also, the use of MRI in diagnostic set up has increased in Finland, similar to what has been previously concluded.^5,7^ The introduction of immunomodulating medications in the mid 1990's might also have affected the number of new diagnoses, since the availability of treatments could encourage neurologists to more avidly seek and set an MS diagnosis. Stabilizing incidence during the last decade could be attributed to decreasing smoking^44^ and improving vitamin D nutrition at the population levels.^45^

Increased prevalence witnessed in our study is likely due to a rise in both incidence and survival. The nationwide prevalence of MS in Finland in 1966 was reported to be 18.6/10^5^.^14^ However, this result was based on pension applications, thus missing mild cases of MS. The study estimated the true prevalence being around 40/10^5^, which is in line with later research from 1975 where prevalence for the city of Helsinki, considered a medium-risk area, was 44.2/10^5^.^14^ Our study shows somewhat lower prevalence, 24.3/10^5^ in 1974–1982. This is most likely due to the more complete population coverage in this study. However, the Finnish regional prevalence studies during the twenty-first century are, more in line with our study, with reported age-adjusted prevalence ranging from 168/10^5^ in Eastern Finland up to 280/10^5^ in Western Finland.^15,22^ We found a prevalence of 218–241/10^5^ in the twenty-first century for both of the McDonald criteria separately, which is similar to previous reports from the other high-risk areas in the world.^9,10,11^

Mean age at the diagnosis was around 40 years in our study, similar to reports in the twenty-first century from other geographical areas,^9,46^ although also markedly lower numbers of around 32 to 37 years have been reported.^6,8,11^ Mean age remained stable throughout the study period. This, combined with increasing incidence, suggests similar pathophysiology captured with the altering diagnostic criteria and a true increase in the MS incidence. The difference between mean age in MS-register compared to the Care register could partly be explained by the demographics of the regions not using MS-register. Care register data had a larger proportion of males, who are more prone to develop primary progressive MS (PPMS).^47,48^ PPMS patients have a higher median age at diagnosis, which could explain the difference.^47,49^ MS-register is a hand-curated database filled by a clinician, whereas Care Register is register-based data and there is some uncertainty considering the method used for identification of pwMS and could thus have a time lag on the date of diagnosis which could further explain the difference.

Increasing incidence and prevalence among women resulted in an increase in the F/M-ratio. This is in line with previous research showing an increase in the F/M-ratio with increased incidence.^3,6,7,50^ Also stable,^12^ or even decreasing ratios have been reported^10^ but in the latter case the F/M-ratio was high, over two during the whole study period, similar to our results. The cause of increasing F/M-ratio is unclear but environmental factors, such as fewer childbirths, increased occurrence of obesity and increased cigarette consumption have been suspected to have a role in this change.^6^

Updates of diagnostic criteria for MS have undoubtedly been shown to enable earlier diagnosis.^32,34,36,37,51,52^ Although the timing of diagnosis was not the topic of this study, a stabilizing trend in the incidence of MS during the twenty-first century seen here may indicate that the current McDonald 2017 criteria just allow for an earlier diagnosis in the patients who would have eventually been diagnosed with earlier McDonald criteria as well, thus not resulting in an increasing, but rather stable incidence. Similar results have previously been seen with the 2005 criteria compared with the 2010 criteria by Runia et al., when the follow-up time was extended to 5 years.^36^

The strength of our study is a population-based dataset, covering the whole nation and with registry data from 1969 onwards. Our limitation is the retrospective study design based on registry data. Care registries have previously shown to have moderate to good (79–93%) ability to identify pwMS.^53,54^ The Finnish Care register for Healthcare has previously been shown to have from satisfactory to very good accuracy and completeness, with 95% of discharges recorded and a positive predictive value between 75 and 95% depending on diagnosis.^55^ Unfortunately, MS validity for the Care Register has to our knowledge not been studied. Results could also be affected by a possible delay in implementation of diagnostic criteria. In the future, it would be of interest to study the most common onset symptom in relation to evolving diagnostic criteria. Also, an analysis of the changing incidence and prevalence rates regarding the east-west gradient on a national level in Finland, with genetic linkage studies, could bring about further understanding on the drivers of increased incidence of MS and especially in females.

In conclusion, our study confirms previous results of Finland being a high incidence and prevalence area for MS. We show a stable incidence during the previous and current McDonald diagnostic criteria and a stable age at diagnosis, both suggesting no major decline in diagnostic accuracy. F-M-ratio shows a drastic increase also in Finland, which is to be investigated further.

## Supplemental Material

sj-docx-1-mso-10.1177_20552173251326173 - Supplemental material for Incidence and prevalence of multiple sclerosis during eras of evolving diagnostic criteria—a nationwide population-based registry study over five decades Supplemental material, sj-docx-1-mso-10.1177_20552173251326173 for Incidence and prevalence of multiple sclerosis during eras of evolving diagnostic criteria—a nationwide population-based registry study over five decades by Anna Maunula, Sini M Laakso, Matias Viitala, Merja Soilu-Hänninen, Marja-Liisa Sumelahti and Sari Atula in Multiple Sclerosis Journal – Experimental, Translational and Clinical

sj-docx-2-mso-10.1177_20552173251326173 - Supplemental material for Incidence and prevalence of multiple sclerosis during eras of evolving diagnostic criteria—a nationwide population-based registry study over five decades Supplemental material, sj-docx-2-mso-10.1177_20552173251326173 for Incidence and prevalence of multiple sclerosis during eras of evolving diagnostic criteria—a nationwide population-based registry study over five decades by Anna Maunula, Sini M Laakso, Matias Viitala, Merja Soilu-Hänninen, Marja-Liisa Sumelahti and Sari Atula in Multiple Sclerosis Journal – Experimental, Translational and Clinical
