# Supplementary material for: Incidence and prevalence of multiple sclerosis during eras of evolving diagnostic criteria—a nationwide population-based registry study over five decades
Source: Mult Scler J Exp Transl Clin. 2025 Mar 16;11(1):20552173251326173. doi: 10.1177/20552173251326173 (PMC11912163; doi:10.1177/20552173251326173)
Supplement: sj-docx-1-mso-10.1177_20552173251326173 - Supplemental material for Incidence and prevalence of multiple sclerosis during eras of evolving diagnostic criteria—a nationwide population-based registry study over five decades [file sj-docx-1-mso-10.1177_20552173251326173.docx]

Supplementary table 1

|  | **Schumacher criteria  (1974-1982) (n=1558)** | **Poser criteria  (1983-2000) (n=5183)** | **Earlier McDonald criteria (2001-2016) (n=7758)** | **Current McDonald criteria  (2017-2021) (n=2276)** |
| --- | --- | --- | --- | --- |
| **Mean age** |  |  |  |  |
| **MS-registry** | 30.4 | 36.2 | 38.6 | 39.9 |
| **Care Register for Healthcare** | 42.6 | 44.4 | 43.9 | 43.5 |
| **Number of pwMS in the cohort from MS-register n (%)** | 391 (25) | 2923 (57) | 6066 (77) | 1833 (80) |
