# Supplementary material for: Incidence and prevalence of multiple sclerosis during eras of evolving diagnostic criteria—a nationwide population-based registry study over five decades
Source: Mult Scler J Exp Transl Clin. 2025 Mar 16;11(1):20552173251326173. doi: 10.1177/20552173251326173 (PMC11912163; doi:10.1177/20552173251326173)

Figure supplement Age-specific incidence per 10^5^ person years by age group at diagnosis divided by sex, shown for different eras of diagnostic criteria.


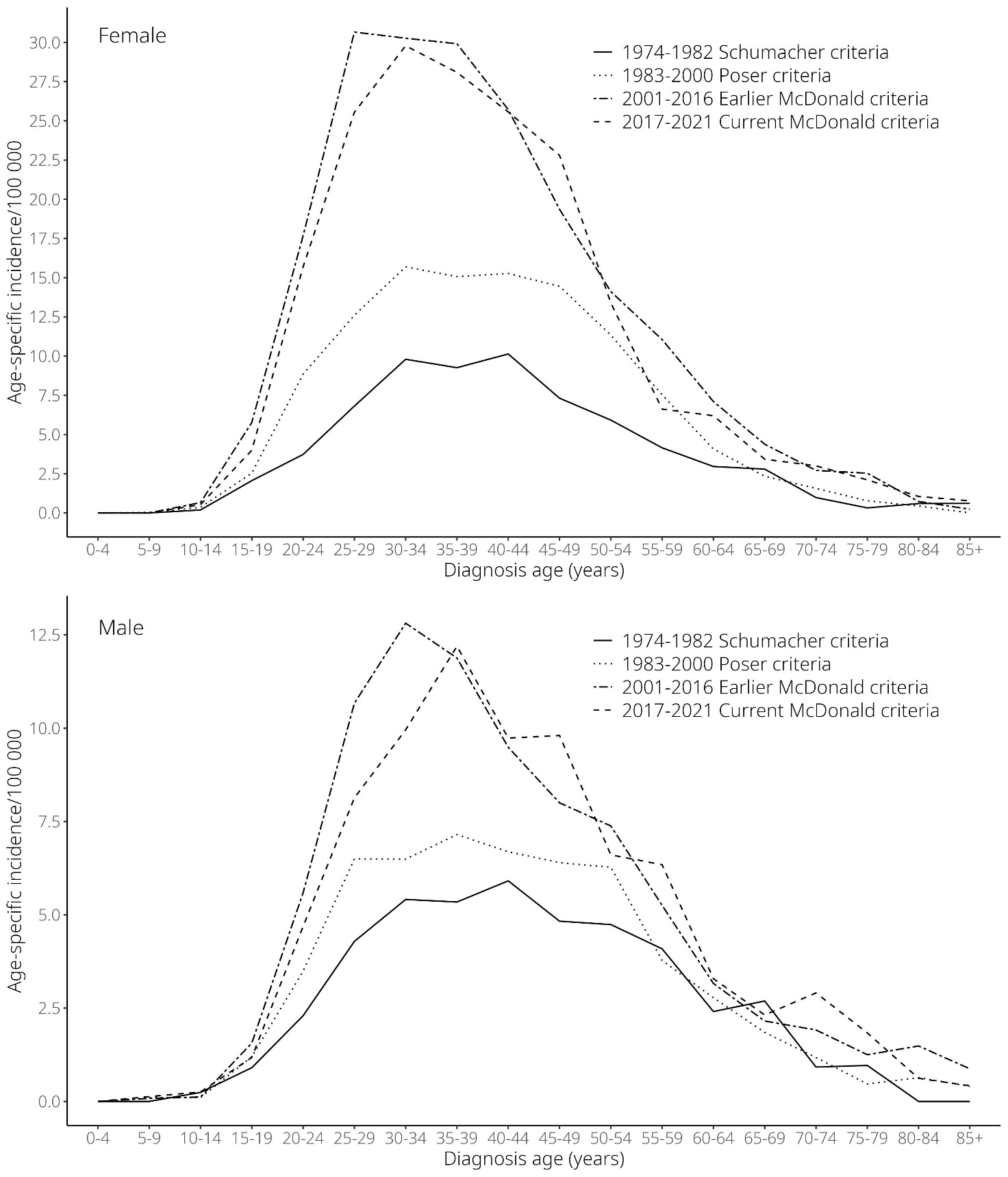

Supplement: sj-docx-2-mso-10.1177_20552173251326173 - Supplemental material for Incidence and prevalence of multiple sclerosis during eras of evolving diagnostic criteria—a nationwide population-based registry study over five decades [file sj-docx-2-mso-10.1177_20552173251326173.docx]
